# A High-Precision Cooperative Localization Method for UAVs Based on Multi-Condition Constraints

**DOI:** 10.3390/s26051641

**Published:** 2026-03-05

**Authors:** Haiqiao Liu, Wen Jiang, Qing Long, Qijun Xia, Xiang Chen

**Affiliations:** 1 The School of Electrical and Information, Hunan Institute of Engineering, Xiangtan 411104, China; xiaqj11@163.com (Q.X.); m15111642499@163.com (X.C.); 2 Graduate School, Hunan Institute of Engineering, Xiangtan 411104, China; jiangwen011017@163.com (W.J.); qinglong1018@163.com (Q.L.)

**Keywords:** cooperative localization, GDOP, formation optimization, GARS, UAV

## Abstract

**Highlights:**

**What are the main findings?**

- Pure air-based swarm configurations suffer from significant *Z*-axis error divergence (3.0–5.0 m) due to insufficient vertical baselines, which cannot be resolved by merely increasing the swarm scale.
- The proposed air–ground cooperative system with edge-deployed ground stations reduces the Position Dilution of Precision (PDOP) to 0.754, effectively suppressing vertical localization drift.

**What are the implications of the main findings?**

- Geometric configuration optimization offers a superior cost–benefit ratio compared to sensor redundancy, proving that the “quality of geometry” outweighs the “quantity of nodes”.
- The established “Stereo Air-Based + Edge Ground-Based” strategy provides a robust engineering paradigm for precise localization in GNSS-denied environments.

**Abstract:**

Global Navigation Satellite Systems (GNSSs) often suffer from significant localization errors in signal-denied environments. Furthermore, the accuracy of multi-UAV cooperative localization is highly sensitive to the relative geometric configuration of the swarm. To address these challenges, this paper proposed a novel high-precision and robust cooperative localization method for UAVs. The proposed method comprised two key modules. First, based on the principle of minimizing the Geometric Dilution of Precision, we optimized both the quantity and geometric configuration of the UAV swarm to identify the top three optimal aerial formations. Second, we introduced Ground-Assisted Reference Stations or Unmanned Ground Vehicles to establish an air–ground cooperative localization system. By leveraging Time Difference of Arrival constraints, this system significantly enhanced localization accuracy and robustness. From this analysis, two optimal hybrid configurations were selected. Experimental results showed that while purely air-based geometric optimization enhanced horizontal coverage, it failed to effectively suppress *Z*-axis errors due to inadequate vertical baselines, with deviations consistently oscillating between 3.0 m and 5.0 m. Conversely, the introduction of edge-deployed ground reference stations reduced the Position Dilution of Precision to a remarkably low level of 0.75, effectively suppressing error divergence. This demonstrated that the proposed air–ground cooperative scheme outperformed traditional pure air-based swarm approaches in localization performance. These findings hold significant theoretical and practical value.

## 1. Introduction

In GNSS-denied environments, such as urban canyons, complex terrains for military reconnaissance, and low-altitude logistics scenarios, high-precision autonomous localization of UAV swarms is a prerequisite for collaborative mission execution. With the large-scale development of the low-altitude economy and its deep integration with IoT technologies, multi-UAV cooperative localization evolved from a singular location service into a comprehensive sensing ecosystem. This ecosystem now encompasses data collection [1], radio environment mapping [2], and large-scale dynamic task scheduling [3]. In these complex scenarios, traditional single-UAV localization was susceptible to multipath effects and signal occlusion. In contrast, cooperative localization technology based on geometric configuration optimization emerged as a critical solution for IoT-assisted networks [4] and terminal relative positioning [5]. By integrating Ultra-Wideband (UWB), visual, and multi-source sensor data, this approach leverages relative observation constraints between nodes to significantly enhance overall system robustness.

Existing research addresses multi-UAV cooperative localization along three primary dimensions: swarm geometric configuration optimization, cooperative strategies under communication constraints, and learning-based data fusion. In the domain of geometric configuration and path planning, optimizing the spatial distribution of UAVs to minimize the GDOP remains a core approach for improving localization accuracy. To operate in complex obstacle-rich environments, ref. [6] introduces rotational potential field terms to balance obstacle avoidance with formation maintenance, while ref. [7] proposes a spatial grid partitioning strategy to satisfy minimum baseline constraints. Building on these foundations, distributed formation control [8] and swarm cooperative localization [9] are further investigated to enhance solution stability by strengthening geometric correlations among nodes. For specific task scenarios, refs. [10,11] explore optimized indoor beacon deployment and fault-tolerant sensor layout, and ref. [12] addresses the single-leader navigation problem under insufficient observation information. However, most of these studies focus primarily on horizontal coverage optimization or the stability of 2D configurations. They largely overlook observation quality in the vertical dimension of 3D space, leading to *Z*-axis localization errors that remain significantly higher than those in the horizontal direction. Moreover, few works quantitatively analyze the marginal cost–benefit trade-off between swarm-scale expansion and localization accuracy gains.

The instability of communication links represented another bottleneck restricting the real-time performance of cooperative localization. Addressing bandwidth constraints and packet loss, refs. [13,14] proposed a hop-limited search model and a data caching retransmission mechanism, respectively, effectively controlling the impact of communication latency on localization accuracy. In terms of dynamic networking, ref. [15] optimized multi-objective communication networks, while refs. [16,17] further explored joint sensing–communication–computing optimization and dynamic deployment strategies under edge-computing architectures to cope with rapid topological changes. Although these studies have significantly enhanced system anti-interference capabilities, in air–ground cooperative scenarios, ground facilities are often treated merely as static communication relays or simple geometric anchors. There was a lack of systematic analysis regarding how the deployment topology of GARS (e.g., edge vs. center distribution) contributes to the GDOP of the overall heterogeneous constellation.

With the advancement of deep learning and multi-modal sensing technologies, data-driven methods provided new perspectives for solving nonlinear localization problems in complex environments. Refs. [18,19] constructed object detection datasets containing multi-class environmental samples and UWB cooperative navigation datasets, respectively, providing benchmarks for algorithm validation. On this basis, refs. [20,21] utilized attention mechanisms and learning-based methods to significantly improve small target detection and visual tracking accuracy. Furthermore, multi-agent reinforcement learning [22] was applied to reliable surveillance tasks, while resonant beam technology [23] and cross-domain cooperative control (air–ground/sea–air) [24,25] have further expanded the physical boundaries of cooperative localization. However, existing learning-based schemes mostly rely on training data from specific scenarios. When facing geometric structural defects such as the lack of vertical baselines, the compensation capability at the pure algorithmic level remains limited.

Due to the nonlinearity and uncertainty in cluster positioning, data-driven and advanced filtering methods have received extensive attention. For instance, ref. [26] proposed using holographic positioning with synthetic reconfigurable intelligent surfaces, achieving extremely high precision at the signal processing level. Similarly, in terms of algorithm robustness, ref. [27] provided a strict integrity monitoring method for particle filtering for mobile robots, emphasizing the trade-off between estimation accuracy and computational safety. Moreover, evolutionary algorithms have also been effectively applied to complex task planning and search tracking, as demonstrated in Ref. [28] in intelligent transportation systems. Particularly crucially, in the physical modeling of measurement errors, recent high-level research has no longer been limited to traditional static noise assumptions. Ref. [29] clearly explored the distance-dependent characteristics of channel noise in wireless sensor networks, verifying the rule that the error variance significantly changes with the increase in physical distance; simultaneously, ref. [30] also proved that positioning uncertainty is essentially driven by the geometric state of the unmanned aerial vehicle. These cutting-edge works fully demonstrate that measurement errors are not constant values, but dynamic variables strictly coupled with spatial distance and topological structure. To effectively mitigate such dynamic measurement anomalies and system uncertainties, recent advancements in state-estimation algorithms have introduced highly robust paradigms. For instance, advanced nonlinear estimators, such as the dual-event-triggering ANFIS-based UKF, have been proposed to uniquely handle measurement anomalies in cluster cooperative navigation [31]. Moreover, since spatial and mathematical constraints are vital for error bounding, advanced constraint-based filtering techniques have drawn significant attention. Recent studies have successfully implemented constrained cubature PF for vehicle navigation [32], hypothesis test-constrained robust Kalman filters for INS/GNSS integration [33], and constrained unscented PF for airship navigation under wind field disturbances [34]. Furthermore, ensuring the reliability of integrated navigation necessitates cutting-edge fault and error tolerance techniques. State-of-the-art mechanisms, including windowing-based factor graph optimization using Mahalanobis distance [35], double-channel sequential probability ratio tests for failure detection [36], and robust UKF frameworks for hypersonic vehicles [37], provide critical anomaly detection capabilities for multi-sensor systems. However, although these advanced nonlinear filters (such as UKF and PF) and complex hardware solutions provide theoretical optimality, they often bring significant computational burdens and delays, which are key constraints for real-time, resource-constrained unmanned aerial vehicle clusters.

In conclusion, although the existing research has made significant progress in configuration control, communication coordination, and advanced signal processing and state estimation, the aforementioned solutions mostly focus on algorithmic compensation. There are still limitations in terms of basic geometric mechanisms and engineering cost-effectiveness. Pure space-based cluster configurations sacrifice vertical observation performance when pursuing horizontal coverage, resulting in *Z*-axis error divergence. Such structural observation blind zones cannot be simply eliminated by filtering algorithms. There is a lack of quantitative assessment of the marginal benefits of the number of drones, computational load, and positioning accuracy. Blindly expanding the cluster size or introducing highly complex estimators will lead to resource redundancy and reduced real-time performance. In the research on air–ground coordination, the geometric layout of ground stations is still unclear regarding the gain mechanism of the overall GDOP of the system.

To address these issues, this paper proposed a Progressive UAV Cooperative Positioning Method based on Multi-condition Constraints (P-UAV-CPM). The main contributions of this paper are as follows:

(1) A geometric optimization model is developed. We established a geometric optimization model for multi-UAV formations. Using GDOP as the indicator, we determined the optimal number of UAVs and identified the top three optimal geometric configurations through simulation, verifying the results using MATLAB R2021b-based simulations.
(2) An air–ground cooperative localization system is designed by integrating ground auxiliary units with TDOA/distance constraints. Corrected geometric precision analysis (PDOP/GDOP) validates the theoretical accuracy improvements, leading to the identification of two optimal hybrid configurations.

The structure of this paper is arranged as follows. Section 1 details the background, significance, and current research status; Section 2 elaborates on the proposed method by modules; Section 3 presents the experimental verification; and the final section concludes the paper.

## 2. Materials and Methods

**Figure 1:**
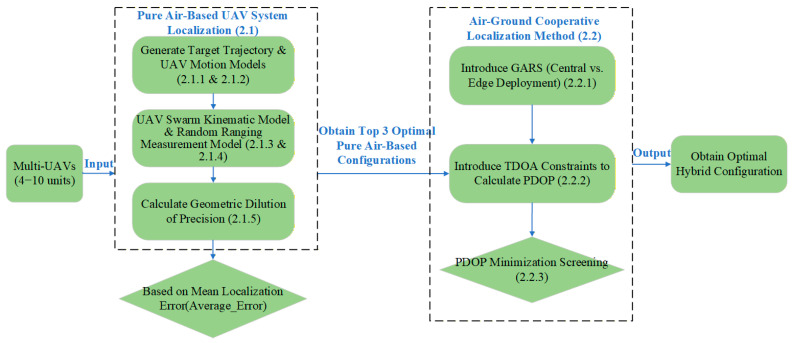
Flowchart of the P-UAV-CPM.

This paper formally defined the motion models of the target and the UAV swarm, as well as the deployment model of GARS within a simulation environment. The objective was to achieve high-precision UAV localization and derive cooperative localization laws. The specific workflow is illustrated in Figure 1.

### 2.1. Pure Air-Based UAV System Localization

#### 2.1.1. Dynamic Target Trajectory Model

To evaluate the robustness of the proposed cooperative localization algorithm in nonlinear complex scenarios, we constructed a 3D dynamic trajectory model based on parameterized harmonic functions. This model utilized a combination of independent parameterized trigonometric functions across three axes to generate motion paths characterized by high smoothness and spatial complexity. The displacement vector of the target at time t, denoted as $\Delta p\left(t\right)$, is defined as follows:

(1) $$\Delta p(t)=\left[\begin{array}{l} \Delta x(t)\\ \Delta y(t)\\ \Delta z(t) \end{array}\right]=\left[\begin{array}{l} A_{x}\sin(B_{x}t+C_{x})\\ A_{y}\cos(B_{y}t+C_{y})\\ A_{z}\sin(B_{z}t+C_{z}) \end{array}\right]$$

where $A_{i},B_{i},C_{i}\left(i\in \left\{x,y,z\right\}\right)$ represents the motion amplitude, angular frequency, and phase offset for each axis, respectively, and t is the discrete sampling time step. By configuring these parameters, the model generated 3D Lissajous-like curves that satisfy kinematic continuity and boundedness constraints. This approach realistically simulates the nonlinear motion characteristics of actual maneuvering targets, thereby establishing a highly dynamic benchmark scenario for validating system tracking accuracy.

The instantaneous absolute position of the target, $p_{\mathrm{target}}\left(t\right)$, was calculated by superimposing the time-varying displacement vector onto the initial position $p_{init}$:

(2) $$p_{\mathrm{target}}(t)=p_{init}+\Delta p(t)$$

#### 2.1.2. UAV Swarm Static Formation Models

The geometric configuration of the UAV swarm directly impacted the accuracy of cooperative localization. This section defined a series of selectable static formations, each possessing distinct geometric properties. All formation models shared the following general parameters: formation center $p_{c}={\left[x_{c},y_{c},z_{c}\right]}^{T}$, radius r, height or interlayer spacing h, and the total number of UAVs N. The position vector of the i-th UAV was denoted as $p_{i}$.

(3) $$p_{i}=\left(\begin{array}{l} x_{c}+r\cos\left(\frac{2\pi i}{N}\right)\\ y_{c}+r\sin\left(\frac{2\pi i}{N}\right)\\ z_{c} \end{array}\right),i=0,1,\ldots ,N-1$$

**Figure 2:**
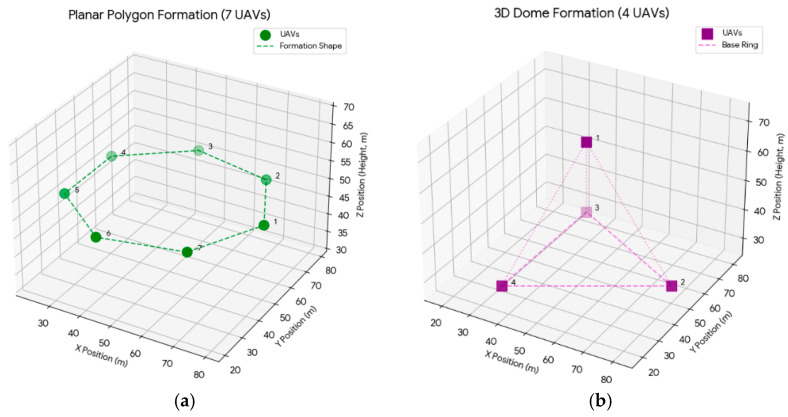

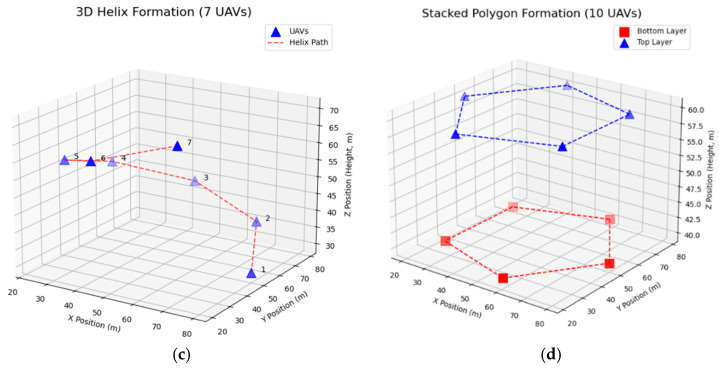
Static formation models of the UAV swarm. (**a**) Planar polygon (7 UAVs); (**b**) 3D Dome (4 UAVs); (**c**) 3D Helix (7 UAVs); (**d**) Stacked Polygon (10 UAVs).

(a) Planar Polygon Formation. The planar polygon formation uniformly distributed all UAVs along a horizontal circumference with radius r centered at $\left(x_{c},y_{c}\right)$. This configuration was suitable for surveillance tasks primarily focused on 2D coverage, as shown in Figure 2a.
  (4) $$p_{i}=\left\{\begin{array}{ll} {\left(x_{c},y_{c},z_{c}+h\right)}^{T} & i=0\\ {\left(x_{c}+r\cos\left(\frac{2\pi (i-1)}{N-1}\right),y_{c}+r\sin\left(\frac{2\pi (i-1)}{N-1}\right),z_{c}-h\right)}^{T} & i=1,\ldots ,N-1 \end{array}\right.$$
(b) 3D Dome Formation. In the 3D Dome formation (requiring N≥4), a hierarchical structure is established. The top UAV supplies a dedicated vertical observation viewpoint, whereas bottom UAVs maintain horizontal coverage. This vertically separated geometric structure was critical for improving the precision of the localization solution, as shown in Figure 2b.
  (5) $$p_{i}=\left(\begin{array}{l} x_{c}+r\cos\left(\frac{2\pi i}{N}\right)\\ y_{c}+r\sin\left(\frac{2\pi i}{N}\right)z_{c}-h+2h\cdot \frac{i}{N-1} \end{array}\right),\;\;\;i=0,1,\ldots ,N-1$$
(c) 3D Helix Formation. The 3D Helix formation (requiring N≥4) arranges UAVs in a spiral pattern along the surface of a virtual cylinder. By offering superior baseline diversity in both horizontal and vertical directions, this configuration theoretically lowers the GDOP and consequently improves localization accuracy, as depicted in Figure 2c.
  (6) pi=xc+rcos2πiN2,yc+rsin2πiN2,zc−hTi=0,…,N2−1xc+rcos2πi−N2N2+πN2,yc+rsin2πi−N2N2+πN2,zc+hTi=N2,…,N−1
(d) Stacked Polygon Formation. The Stacked Polygon formation (requiring N to be an even number and N≥4) consisted of two interlaced planar polygons positioned one above the other. This configuration enhanced the robustness of the geometric structure by adding vertical dimensionality and an interleaved layout, as shown in Figure 2d.

The motivation for introducing 3D formations (such as Dome, Helix, and Stacked Polygon) lay in improving the observability of the target’s vertical position. Compared to pure planar formations, 3D formations provide significant vertical baselines. This was essential for precisely resolving the target’s Z-coordinate, effectively reducing the GDOP value and improving overall localization accuracy. Under this evaluation framework, it must be clearly stated that this study employs kinematic simulation rather than dynamic control simulation. The position deviations during the formation maintenance process are modeled as Gaussian noise, aiming to represent the residual steady-state error after the closed-loop stability of the underlying flight controller (such as PID). This abstraction and simplification of the dynamic process isolate the transient interference of specific control law parameters, allowing for a more focused quantitative analysis of the pure contribution of the three-dimensional geometric configuration to positioning accuracy. The specific quantitative results will be elaborated in the subsequent sections.

#### 2.1.3. UAV Swarm Kinematic Model

This section describes how the static formations evolved spatially over time. The model assumed that the swarm operated as a rigid body, where its geometric center (centroid) perfectly tracked the target’s trajectory. The position of the swarm centroid updated as follows:

(7) $$p_{centroid}\left(t\right)=p_{centroid}\left(0\right)+\left(p_{\mathrm{target}}\left(t\right)-p_{\mathrm{target}}\left(0\right)\right)$$

where $p_{centroid}\left(0\right)$ was the centroid’s position at the initial time. This equation implied that the global displacement of the swarm aligned perfectly with that of the target. Based on the moving centroid, the ideal position of the i-th UAV was calculated as follows:

(8) $$p_{ideal,i}\left(t\right)=p_{centroid}\left(t\right)+\left(p_{uav,i}\left(0\right)-p_{centroid}\left(0\right)\right)$$

This formula ensured that the initial offset vector of each UAV relative to the swarm centroid remained constant, thereby maintaining the initially defined formation structure under ideal conditions. However, in practical applications, UAVs have been observed to experience difficulties in maintaining their relative positions with absolute precision. To simulate this uncertainty, formation keeping noise was introduced. The final simulated position of the i-th UAV was obtained by superimposing a zero-mean 3D Gaussian random noise vector onto its ideal position:

(9) $$p_{uav,i}\left(i\right)=p_{ideal,i}\left(t\right)+\eta ,\;\;\;\;\eta ∼N\left(0,\sigma_{formation}^{2}I\right)$$

where $\sigma_{formation}$ was the standard deviation of the formation keeping noise, and I was the 3×3 identity matrix. This noise term accounts for small positional perturbations arising from control inaccuracies and environmental disturbances such as wind. Notably, the kinematic model adopts an idealized open-loop control assumption, wherein the swarm possesses perfect knowledge of the target’s true displacement. This enables an isolated evaluation of the localization algorithm, free from the confounding effects of closed-loop tracking dynamics.

#### 2.1.4. Random Ranging Measurement Model

To simulate the uncertainties in real-world environments, this paper employs a simplified linear superposition noise model. Although this model does not involve complex physical modeling of the signal-to-noise ratio, it aims to capture the effects of distance attenuation and multi-node interference on the accuracy of ranging. For the i-th UAV, the true Euclidean distance to the target at time t was as follows:

(10) $$d_{true,i}\left(t\right)=\left|p_{uav,i}\left(t\right)-p_{\mathrm{target}}\left(t\right)\right|$$

The standard deviation of the total measurement noise was a linear superposition of three components. $\sigma_{base}=0.5\;m$ represents the basic system error of the sensor; $k_{dist}=0.01$ represents the distance influence factor, simulating the attenuation trend of the analog signal; and $k_{\mathrm{int}erf}=0.1\;m/UAV$ represents the multi-vehicle interference coefficient, indicating the additional signal crosstalk noise introduced by each additional unmanned aircraft when the cluster size exceeds 4 aircraft:

(11) $$\sigma_{total,i}\left(t\right)=\sigma_{base}+d_{true,i}\left(t\right)\cdot k_{dist}+\max\left(0,N-N_{base}\right)\cdot k_{\mathrm{int}erf}$$

#### 2.1.5. GDOP

The GDOP was a critical performance metric that quantified the impact of the observed geometric configuration on localization accuracy, independent of the magnitude of measurement noise. Firstly, we defined the geometry matrix (also referred to as the Jacobian matrix), denoted as H. Each row of this matrix corresponded to the unit line-of-sight (LOS) vector, with the orientation of this vector pointing from the target position to the i-th UAV:

(12) $$H=\left[\begin{array}{l} u_{1}^{T}\\ M\\ u_{N}^{T} \end{array}\right],\;\;\;u_{i}=\frac{p_{uav,i}-p_{target}}{\left\|p_{uav,i}-p_{target}\right\|}$$

The GDOP was calculated using the following formula:

(13) $$GDOP=\sqrt{trace\left({\left(H^{T}H\right)}^{-1}\right)}$$

where $trace\left(\cdot \right)$ denoted the matrix trace operator. GDOP was a dimensionless metric that characterized the magnification factor by which measurement noise was translated into localization error. A lower GDOP value indicated that the swarm possessed a robust spatial configuration and error suppression capability. Conversely, a high GDOP value typically corresponded to “ill-conditioned geometric distributions,” such as multi-UAV collinearity. In such cases, the system operates on the verge of geometric singularity, rendering the localization solution extremely sensitive to ranging noise.

### 2.2. UAV System Localization Under Ground Constraints

#### 2.2.1. Deployment Model of GARS

This model defined the specific coordinates of fixed ground sensors on the map, with the aim of systematically analyzing the impact of the quantity and location of GARS on the localization accuracy of the entire hybrid system. Let the center of the GARS deployment area be $\left(cx,cy\right)$, the semi-width be hw, and the semi-depth be hd.

Central Deployment: The GARS were deployed intensively near the geometric center of the measurement area, forming a relatively compact ground reference network.

(14) $$N_{gars}=1:P=\left[cx,cy,0\right]$$

(15) $$N_{gars}=2:P=\left\{\left[cx-15,cy,0\right],\left[cx+15,cy,0\right]\right\}$$

(16) $$N_{gars}=3:P=\left\{\left[cx,cy+15,0\right],\left[cx-15,cy-15,0\right],\left[cx+15,cy-15,0\right]\right\}$$

Edge Deployment: The GARS were deployed in a dispersed manner at the edges or corners of the measurement area. This strategy aimed to maximize the baseline length between ground sensors, thereby providing stronger and broader geometric constraints for the TDOA solution.

(17) $$N_{gars}=2:P=\left\{\left[cx-hw,cy-hd,0\right],\left[cx+hw,cy+hd,0\right]\right\}$$

(18) $$N_{gars}=3:P=\left\{\left[cx-hw,cy-hd,0\right],\left[cx+hw,cy-hd,0\right],\left[cx,cy+hd,0\right]\right\}$$

With the introduction of GARS, this paper has constructed a highly robust TOA/TDOA hybrid cooperative positioning architecture. The design of this hybrid system is mainly based on engineering feasibility considerations: unlike the highly dynamic UAV clusters, the fixed-position ground stations can achieve high-precision clock synchronization through wired links, making the TDOA system without two-way interaction possible, significantly reducing communication overhead, while the cluster still retains the TOA bidirectional ranging that is insensitive to clock deviations to maintain the relative configuration. Mathematically, this transformation means that the geometric intersection model changes from the “sphere intersection” of TOA to the “hyperboloid intersection” of TDOA. Therefore, in order to rigorously evaluate the geometric observation quality of this hybrid heterogeneous system, the core performance indicator is correspondingly adjusted from the general GDOP to the TDOA-PDOP, which is derived specifically based on the TDOA differential observation matrix, to more accurately quantify the suppression effect of ground constraints on the vertical direction error.

#### 2.2.2. TDOA Geometry Matrix (H)

The calculation of TDOA-PDOP was based on the geometry matrix H. The geometric relationship between all sensors (GARS and UAVs) and the target was mathematically modeled in this study, according to the principles of TDOA. Calculating TDOA required a reference sensor (in the simulation code, a GARS was selected as the reference). Unit Line-of-Sight (LOS) Vectors: Let the target position be $P_{T}$. For the reference sensor ($d_{0}=\left\|P_{T}-P_{0}\right\|$),

(19) $$u_{0}=\frac{P_{T}-P_{0}}{d_{0}}$$

For other sensors (Sensor i), $d_{i}=\left\|P_{T}-P_{i}\right\|$

(20) $$u_{i}=\frac{P_{T}-P_{i}}{d_{i}}$$

Construction of the H Matrix. The mathematical essence of TDOA was embodied in the construction of the H matrix. Each row of the matrix was obtained by subtracting the LOS vector of the reference sensor 0 from that of sensor i. This was a direct mapping of the TDOA (time difference) concept onto geometric space:

(21) $$\begin{array}{c} h_{i}=u_{i}-u_{0}\\ H=\left[\begin{array}{c} h_{1}\\ M\\ h_{N-1} \end{array}\right]=\left[\begin{array}{c} u_{1}-u_{0}\\ M\\ u_{N-1}-u_{0} \end{array}\right] \end{array}$$

#### 2.2.3. PDOP Calculation

Covariance Matrix. The covariance matrix of the position estimation error was obtained through linear algebra operations:

(22) $$Q={\left(H^{T}H\right)}^{-1}$$

The diagonal elements of this matrix quantified the magnification degree (i.e., variance) of the localization error along the X, Y, and Z axes, respectively.

PDOP Value. The PDOP value was calculated from the trace of the matrix Q. This operation summed the variances of the three axes, representing the total error variance in 3D space, and finally took the square root to convert it into distance units:

(23) $$PDOP=\sqrt{trace\left(Q\right)}$$

## 3. Results

### 3.1. Experimental Setup

Experimental Environment. The simulation was implemented in Python 3.13, utilizing the PyCharm 2024 Integrated Development Environment (IDE).

Simulation Scenario. The operational environment was simulated within a defined spatial region (e.g., a regular polygon or volume of size 500×500×100 m). The target was initially positioned at the center of this volume or distributed randomly within it. Each configuration was tested over 300 discrete time steps along random trajectories, with the objective of evaluating its localization performance.

To statistically evaluate the simulation results, the following metrics were adopted: To calculate the estimated position $\hat{p_{k}}$, a linear least squares algorithm based on the sum of squared distances was adopted. Suppose there are N UAV nodes with known positions. The position of the i node was $p_{i}$ and the measurement distance was $d_{i}$. Eliminate the nonlinear quadratic terms in the ranging equation, subtract the ranging equation of the i node (i=1,…,N−1) from that of the 0th reference node, and thus construct the following linear equation system:

(24) $$A\hat{p_{k}}=b$$

Among them, the i row of the geometric matrix A was $2{\left(p_{i}-p_{0}\right)}^{T}$, and the i element of the observation vector b was $d_{0}^{2}-d_{i}^{2}+{\left\|p_{i}\right\|}^{2}-{\left\|p_{0}\right\|}^{2}$. The final estimated position $\hat{p_{k}}$ was obtained through LS solution:

(25) $$\hat{p_{k}}={\left(A^{T}A\right)}^{-1}A^{T}b$$

(1) Instantaneous Localization Error ($Error_{k}$). At the k-th time step, the localization error was defined as the Euclidean distance between the estimated position $\hat{p_{k}}$ and the ground truth position $p_{true,k}$:
  (26) $$Error_{k}=\left|\hat{p_{k}}-p_{true,k}\right|$$
(2) Mean Localization Error ($\bar{E}$). The arithmetic mean of the instantaneous errors over all valid time steps ($N_{valid}$), used to measure the overall accuracy or bias of the localization results, is as follows:
  (27) $$\bar{E}=\frac{1}{N_{valid}}\sum_{k\in valid\;steps}Error_{k}$$
(3) Standard Deviation of Error ($S_{E}$). The sample standard deviation of the localization errors for all successful steps, used to measure the dispersion or stability (precision) of the results, is as follows:
  (28) $$S_{E}=\sqrt{\frac{1}{N_{valid}-1}{\sum_{k\in valid\;steps}\left(Error_{k}-\bar{E}\right)}^{2}}$$
(4) Localization Success Rate ($R_{success}$). The percentage of valid localization attempts ($N_{valid}$) relative to the total number of simulation steps ($N_{total}$), measuring the algorithm’s reliability and robustness, is calculated as follows:
  (29) $$R_{success}=\frac{N_{valid}}{N_{total}}\times 100\%$$

### 3.2. Results and Discussion of Pure Air-Based UAV System Localization

The experimental simulation results are shown in Table 1. This table compares the positioning performance of each configuration under different cluster sizes. Through the numerical comparison of mean error and the analysis of abnormal states, the data reveals significant performance differentiation and provides a key basis for subsequent configuration optimization: Firstly, the planar configuration fails in positioning due to geometric defects. The data shows that the P-Polygon configuration with coplanar distribution exhibits extreme instability, especially when N=6, its standard deviation increases to 432.98 m, and the mean error reaches 168.98 m. This is attributed to the inherent vertical singularity of coplanar geometry. Due to the lack of a vertical baseline, the observation matrix is close to rank deficiency in the *Z*-axis direction, resulting in the least squares solution being extremely sensitive to ranging noise, thereby causing significant position drift. This proves that a purely planar formation cannot meet the requirements of high-reliability positioning. Secondly, for three-dimensional configurations, the cluster size is not necessarily the larger the better. Taking the Dome as an example, when N increases from 4 to 10, the positioning error deteriorates from 1.60 m to 9.56 m. This reveals the influence of “geometric gain” and “signal interference”. Although the increase in nodes improves the theoretical GDOP, the signal congestion caused by high density (Formula 11) introduces excessive ranging noise, offsetting the geometric benefits brought by the formation. The same situation occurs with the Helix, which maintains an error of 2.87 m at N=10; although it is better than the deteriorated Dome, it is still slightly inferior to the layered structure of S-Polygon.

In conclusion, based on the quantitative screening using mean error, the three most representative configurations were identified from Table 1 as the focus of subsequent research: 4UAVS (3D Dome), 10UAVS (Stacked Polygon), and 4UAVS (Stacked Polygon).

**Figure 3:**
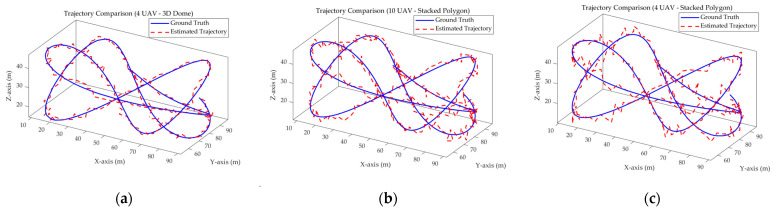
3D trajectory tracking results. (**a**–**c**) depict the localization paths for different configurations.

Figure 3 visualizes the trajectory tracking performance for the three representative configurations along a randomized path of uniform parameters, derived from Formula (1) and Formula (25). By setting randomized start and end points and irregular routes, the test rigorously challenged the localization capability of each system. Figure 3a,b highlight the impact of formation geometry under identical swarm sizes (4 UAVs). Conversely, a comparison between Figure 3b,c and Table 1 revealed that increasing the number of UAVs did not necessarily yield proportional accuracy gains.

**Figure 4:**
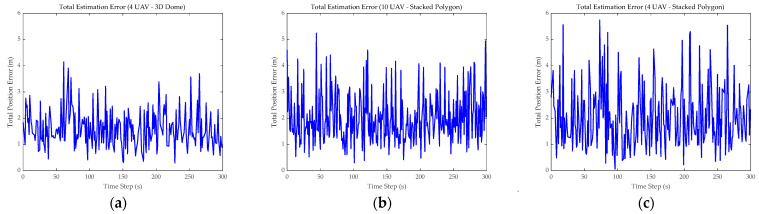
Error analysis. (**a**–**c**) show the fluctuation of total localization error for different combinations.

Figure 4 compares the fluctuation of the total localization error over the simulation cycle for the three typical combinations, as derived from Formula (26). The results indicated that the quality of the geometric configuration had a far greater impact on accuracy than the mere accumulation of sensor quantity. First, with a consistent swarm size of four UAVs, the error peak of the 3D Dome configuration (Figure 4a) was effectively controlled at approximately 4.2 m, exhibiting relatively convergent waveforms. In contrast, the Stacked Polygon configuration (Figure 4c) showed error peaks exceeding 5.5 m, accompanied by high-frequency oscillations. This significant discrepancy demonstrated that the magnitude of the vertical geometric aperture was the primary factor determining system robustness. A comparison between Figure 4b,c reveals that increasing the number of UAVs in the Stacked Polygon formation from four to ten yields only a marginal reduction in the peak error—from 5.6 m to 5.2 m—failing to achieve the anticipated accuracy improvement. This phenomenon confirmed the hypothesis proposed earlier: merely increasing the number of UAVs may not improve localization accuracy. In the absence of configuration optimization, the blind addition of nodes introduces measurement redundancy, while the accompanying signal crosstalk noise ($\sigma_{int}$) offsets most of the geometric gains.

**Figure 5:**
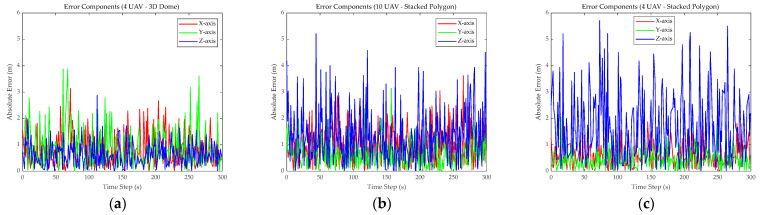
Axial error analysis. (**a**–**c**) show the independent axial errors for X, Y, and Z axes for different combinations.

To further investigate the sources of error, Figure 5 decouples the total error into three independent axial components (X, Y, Z):

(30) $$\left\{\begin{array}{l} E_{x,k}=\left|\hat{x_{k}}-x_{true,k}\right|\\ E_{y,k}=\left|\hat{y_{k}}-y_{true,k}\right|\\ E_{z,k}=\left|\hat{z_{k}}-z_{true,k}\right| \end{array}\right.$$

As demonstrated in Figure 5b (10 UAVs) and Figure 5c (four UAVs), a pronounced “anisotropy” in the error of the Stacked Polygon configuration was evident. The blue curve, representing the *Z*-axis error, exhibited a dominant influence on the error profile, maintaining a high amplitude between 3.0 m and 5.0 m for extended periods. In contrast, the horizontal errors (X/Y axes) generally remained below 1.5 m. This phenomenon, where the vertical error was two to three times the horizontal error, intuitively revealed the geometric essence of the configuration’s excessive Vertical Dilution of Precision (VDOP)—namely, the lack of vertical geometric support caused by “large distances and short baselines.” Conversely, the 3D Dome configuration in Figure 5a exhibited a relatively balanced error distribution across all three axes. Although the *Y*-axis error (green line) occasionally spiked (peak approx. 3.5 m) in certain trajectory segments, the most challenging *Z*-axis error (blue line) was successfully suppressed within the 2.0 m to 2.5 m range. This provided ample evidence that a stereo-geometric structure constructed by extending vertical baselines can effectively compensate for observability defects in a single direction, thereby achieving balanced localization in 3D space.

**Figure 6:**
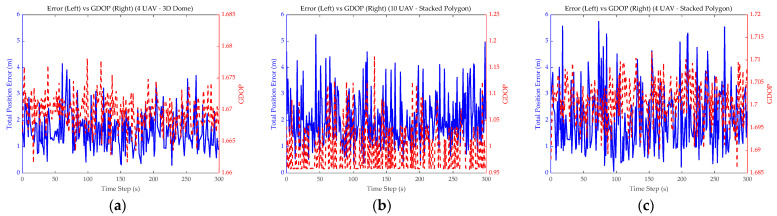
GDOP vs. error variation. (**a**–**c**) show the variation in GDOP and error over time for different combinations.

Figure 6 illustrates the variation in the GDOP over time and error. It is evident from Figure 6a that the GDOP variation for the 3D Dome was minimal and nearly constant; the fluctuations in error were therefore likely attributable to non-geometric factors such as ranging noise, UAV motion model errors, and estimation filter noise. Figure 6b shows that increasing the UAV count to 10 significantly reduced the GDOP, yet it failed to effectively suppress the fluctuations and peaks of the total error. The reason was likely consistent with the analysis above (Figure 4b,c). Figure 6c shows a GDOP distribution similar to Figure 6a, but due to differing geometric influences within the formations, the resulting errors varied drastically. This further illustrates the defects of the Stacked Polygon formation.

**Figure 7:**
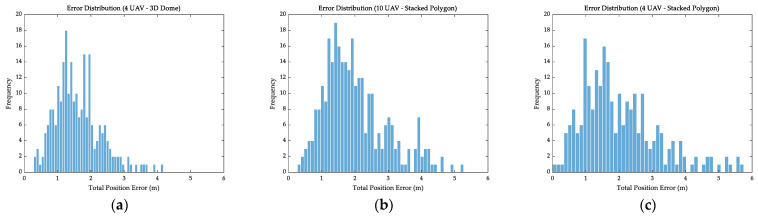
Error frequency distribution. (**a**–**c**) show the frequency of occurrence for specific errors for different combinations.

Figure 7 depicts the frequency distribution of specific error magnitudes. It could be seen that Figure 7a (3D Dome) performed best, with the most compact and concentrated error distribution. The remaining two configurations exhibited distinctly wider and flatter distributions. This indirectly verified that the limitations and structural defects of the Stacked Polygon formation outweigh the advantages of numerical scale.

### 3.3. Results and Discussion of UAV System Localization Under Ground Constraints

**Figure 8:**
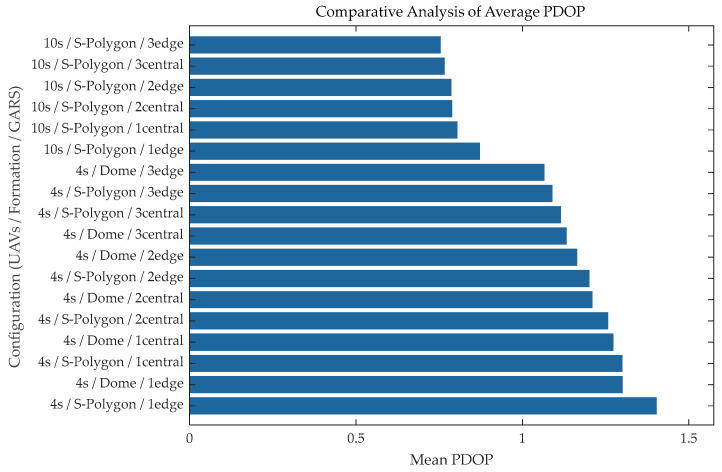
Localization results of the UAV system under ground constraints.

To effectively mitigate the localization drift of pure UAV swarms in the vertical direction, a GARS-based air–ground cooperative enhancement scheme was proposed. The fundamental rationale underlying this investigation was to ascertain the influence of the geometric configuration of ground baselines, with particular reference to the quantity and deployment location (central vs. edge) of GARS, on the overall geometric strength of the system. By comparing the PDOP of various “air–ground” hybrid combinations, we aimed to identify the optimal configuration capable of maximizing geometric gain. As illustrated in Figure 8, the specific simulation comparison results are presented, detailing the mean PDOP performance of each hybrid configuration along the simulated trajectory.

Experimental data revealed that the “10-UAV Stacked Polygon + 3 Edge GARS” configuration exhibited the optimal geometric strength among all tested combinations. The mean PDOP of this configuration converged to 0.754, which was not only significantly lower than similar configurations with four-UAV swarms (whose PDOP values are generally above 1.0) but also outperformed the centrally deployed (central) schemes. This result quantitatively confirmed that, within the context of an air–ground cooperative system, augmenting the swarm scale and adopting an edge-deployed ground station strategy were efficacious in maximally reducing the GDOP, thereby providing the most robust geometric support for high-precision localization.

## 4. Discussion

**Figure 9:**
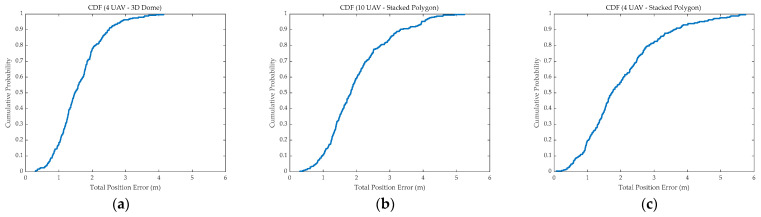
Cumulative Distribution Function (CDF) of error. (**a**–**c**) show curves for different combinations.

**Figure 10:**
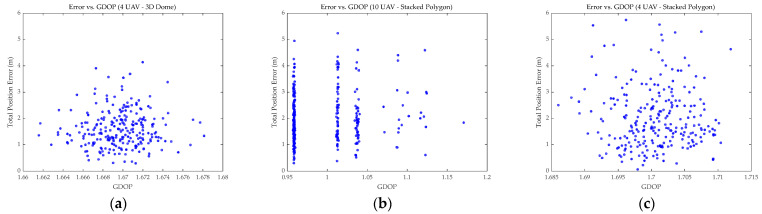
Scatter plots. (**a**–**c**) show the correlation between total localization error and GDOP for different combinations.

To reveal the inherent logic of the progressive evolution from “pure air-based geometric optimization” to “air–ground heterogeneous cooperation,” this section provides a comprehensive discussion combining the statistical characteristics of the Cumulative Distribution Function (CDF) of error (Figure 9) and the Error-GDOP scatter plot (Figure 10). Emphasis was placed on the analysis of the manner in which the second phase specifically overcame the physical bottlenecks of the first phase.

(1) Analysis of limitations in pure air-based geometric optimization. Experimental results indicated that the localization accuracy of pure air-based schemes was limited by the physical envelope of the swarm geometric configuration, rather than being solely dependent on the node scale. Comparing Figure 4 and Figure 5, it was evident that while the widely used Stacked Polygon configuration offered excellent horizontal coverage; its insufficient interlayer baselines lead to a lack of geometric support in the *Z*-axis, resulting in *Z*-axis errors persisting in the high range of 3.0 m to 5.0 m. Crucially, the scatter distribution in Figure 10 revealed significant “diminishing marginal returns”: even when the number of UAVs was increased from 4 to 10, the distribution pattern of the GDOP did not fundamentally change, and the error oscillation in the vertical direction was not eliminated. This confirmed that in the absence of effective vertical baselines, merely augmenting the redundancy of homogeneous sensors could not adequately compensate for structural geometric defects.
(2) Verification of advantages in air–ground cooperative enhancement. It is also worth noting the comparison with advanced signal processing and filtering techniques mentioned in the Introduction. While methods such as PF and Variational Bayesian Learning can effectively smooth trajectory noise, they rely on the premise that the system is fully observable. In the “Stacked Polygon” formation, the *Z*-axis error divergence stems from a structural singularity characterized by an excessive VDOP rather than simple random measurement noise. As demonstrated by the analysis, positioning uncertainty is fundamentally driven by the geometric state. Therefore, without the physical constraints provided by the GARS, purely algorithmic compensation faces a high risk of divergence. The proposed air–ground strategy addresses the geometric defect as the root cause of the error, thereby establishing a robust foundation for any subsequent algorithmic processing. In contrast, the air–ground cooperative scheme introducing GARS fundamentally reshaped the system’s observation geometry. In the context of adopting the “Edge Deployment” strategy, ground sites and the aerial swarm were found to impose heterogeneous constraints with long baselines, thereby effectively closing the originally open vertical error channel. As described in Section 3.3, this scheme successfully converged the PDOP value of the optimal configuration to 0.754. This qualitative leap proved that optimizing spatial geometric distribution by introducing heterogeneous nodes could break through the accuracy bottleneck of pure air-based swarms at a lower resource cost, achieving a better cost-effectiveness ratio than merely piling up sensor quantities.
(3) Cost–Benefit analysis and future directions. As demonstrated by Figure 8, after effectively overcoming the observation bottleneck in the vertical dimension, the 10-UAV Stacked Polygon formation was significantly superior to other formations. However, from the perspective of practical economic costs, despite the addition of six UAVs, the PDOP value of the lowest combination was only approximately 30% higher than that of the 4UAVS_Stacked_Polygon combination. Therefore, considering practical costs and other factors, the combinations “10UAVS_Stacked_Polygon” and “4UAVS_Stacked_Polygon” combined with “3GARS_edge” should be prioritized in future research to investigate optimal trade-offs. It should be noted that the state estimation in our current simulation framework employs a linear LS estimator to solve the nonlinear spherical intersection equations. While the LS approach is mathematically straightforward and effectively isolates the pure impact of spatial geometric variables without introducing algorithmic compensation biases, it inevitably suffers from linearization errors, particularly under high measurement noise conditions. In practical, highly nonlinear UAV cooperative navigation scenarios, these linearization errors must be systematically mitigated. As suggested by recent advancements, advanced nonlinear filtering techniques, such as the UKF, CKF, and PF, possess robust capabilities to handle such linearization errors and measurement uncertainties. The geometric optimization proposed in this study establishes a robust spatial baseline (e.g., reducing the PDOP to 0.754); therefore, integrating this optimized air–ground configuration with state-of-the-art nonlinear filters (e.g., UKF, CKF, or PF) constitutes a critical direction for our future work.

## 5. Conclusions

Addressing the accuracy bottlenecks of multi-UAV cooperative localization in GNSS-denied environments, this paper proposes a method based on geometric configuration evolution and air–ground cooperative enhancement. The research first reveals the physical limitations of pure air-based swarms: localization accuracy is nonlinearly correlated with the number of nodes. The widely used “Stacked Polygon” configuration, due to insufficient vertical baselines, suffers from significant divergence in *Z*-axis error, whereas the “3D Dome” configuration, with its superior volumetric envelope, exhibits greater robustness at the same scale. The following research is an extension of the preceding study on an air–ground cooperative architecture incorporating GARS, confirming the suppression effect of heterogeneous geometric constraints on error divergence. Simulation results demonstrate that adopting the “Edge Deployment” strategy effectively compensates for vertical observation blind spots, with the “10-UAV Stacked Polygon + Edge GARS” hybrid configuration successfully causing the *Z*-axis error to converge to sub-meter levels. This study ultimately establishes an optimization strategy of “Stereo Air-Based + Edge Ground-Based,” demonstrating that rational geometric distribution design offers a superior cost-effectiveness ratio compared to mere sensor redundancy. This provides a reliable engineering paradigm for high-precision swarm localization in complex environments.

## Figures and Tables

**Table 1:** Quantitative comparison of positioning performance under different formation configurations and swarm scales.

Swarm Size (*N*)	Configuration	Mean Error (m)	Mean GDOP	Std Error	Success Rate (%)
**10**	**P-P** **oly** **gon**	**66.69**	**0.99**	**65.48**	**100** **.00**
8	P-Polygon	73.08	1.15	73.38	100.00
9	P-Polygon	74.69	1.07	73.88	100.00
7	P-Polygon	97.41	1.26	90.59	100.00
6	P-Polygon	168.98	1.43	432.98	100.00
**4**	**D** **ome**	**1.60**	**1.66**	**0.68**	**81** **.00**
9	Dome	6.87	1.26	36.19	100.00
10	Dome	9.56	1.24	37.21	100.00
8	Dome	10.02	1.30	45.66	100.00
5	Dome	16.60	1.50	135.45	98.00
7	Dome	16.73	1.36	83.26	100.00
6	Dome	29.21	1.43	337.15	100.00
**9**	**H** **elix**	**2.78**	**1.12**	**1.62**	**100** **.00**
7	Helix	2.86	1.35	2.09	100.00
10	Helix	2.87	1.05	1.72	100.00
8	Helix	2.89	1.22	1.83	100.00
6	Helix	2.97	1.53	2.40	99.67
4	Helix	3.23	1.87	2.04	83.33
5	Helix	3.26	1.72	2.69	98.00
**10**	**S** **-** **Poly** **gon**	**2.00**	**0.99**	**0.94**	**100** **.00**
**4**	**S** **-** **Poly** **gon**	**2.01**	**1.70**	**1.15**	**82.33**
8	S-Polygon	2.02	1.15	1.05	100.00
6	S-Polygon	2.51	1.38	8.12	100.00

**Note:** Bold text indicates the minimum GDOP achieved within each formation type and highlights the three optimal combinations selected for subsequent evaluations.

## Data Availability

Data available on request due to restrictions on privacy.
